# Dichotomous Distribution of Putative Cholinergic Interneurons in Mouse Accessory Olfactory Bulb

**DOI:** 10.3389/fnana.2017.00010

**Published:** 2017-02-27

**Authors:** Sarah Marking, Kurt Krosnowski, Tatsuya Ogura, Weihong Lin

**Affiliations:** Department of Biological Sciences, University of Maryland, Baltimore CountyBaltimore, MD, USA

**Keywords:** olfactory system, accessory olfactory bulb, vomeronasal organ, cholinergic, vesicular acetylcholine transporter, choline acetyltransferase, olfactory glomeruli

## Abstract

Sensory information processing in the olfactory bulb (OB) relies on diverse populations of bulbar interneurons. In rodents, the accessory OB (AOB) is divided into two bulbar regions, the anterior (aAOB) and posterior (pAOB), which differ substantially in their circuitry connections and associated behaviors. We previously identified and characterized a large number of morphologically diverse cholinergic interneurons in the main OB (MOB) using transgenic mice to visualize the cell bodies of choline acetyltransferase (ChAT-expressing neurons and immunolabeling (Krosnowski et al., 2012)). However, whether there are cholinergic neurons in the AOB is controversial and there is no detailed characterization of such neurons. Using the same line of ChAT^(bacterial artificial chromosome, BAC)^-enhanced green fluorescent protein (eGFP) transgenic mice, we investigated cholinergic neurons in the AOB. We found significant differences in the number and location of GFP-expressing (GFP+), putative cholinergic interneurons between the aAOB and pAOB. The highest numbers of GFP+ interneurons were found in the aAOB glomerular layer (aGL) and pAOB mitral/tufted cell layer (pMCL). We also noted a high density of GFP+ interneurons encircling the border region of the pMCL. Interestingly, a small subset of glomeruli in the middle of the GL receives strong MCL GFP+ nerve processes. These local putative cholinergic-innervated glomeruli are situated just outside the aGL, setting the boundary between the pGL and aGL. Many but not all GFP+ neurons in the AOB were weakly labeled with antibodies against ChAT and vesicular acetylcholine transporter (VAChT). We further determined if these GFP+ interneurons differ from other previously characterized interneuron populations in the AOB and found that AOB GFP+ interneurons express neither GABAergic nor dopaminergic markers and most also do not express the glutamatergic marker. Similar to the cholinergic interneurons of the MOB, some AOB GFP+ interneurons express the calcium binding protein, calbindin-D28K. Moreover, exposure to either a male intruder or soiled bedding from a mating cage leads to an increase in the number of c-Fos-expressing MCL GFP+ neurons. Taken together, our data reveal a population of largely unidentified putative cholinergic neurons in the AOB. Their dichotomous distribution in the aAOB and pAOB suggests region-specific cholinergic involvement in olfactory information processing.

## Introduction

The olfactory bulb (OB) is the first brain region receiving and processing olfactory sensory input (Shipley and Ennis, 1996; Nagayama et al., 2014). In rodents, the OB is anatomically and functionally divided into the main and accessory OBs (MOB and AOB, respectively), with the AOB occupying the posterodorsal-middle region (Mori et al., 1999). The AOB is an integral part of the accessory olfactory system (AOS), which processes sensory signals from the vomeronasal organ (VNO; Halpern and Martínez-Marcos, 2003; Mucignat-Caretta, 2010; Pérez-Gómez et al., 2014). The distinct evoked patterns of AOB neuron activity is known to encode sexual and genetic statuses of the conspecific donor animals and identities of predators (Luo et al., 2003; Ben-Shaul et al., 2010; Tolokh et al., 2013; Pérez-Gómez et al., 2014). AOB output to the hypothalamic nuclei leads to specific social and reproductive behaviors and endocrine responses, such as mating, aggression and innate fear reaction to predators (He et al., 2008; Rodriguez and Boehm, 2009; Tirindelli et al., 2009; Ben-Shaul et al., 2010; Papes et al., 2010; Chamero et al., 2011; Pérez-Gómez et al., 2014). However, knowledge of local interneuron networks involving information processing in the AOB is very limited.

Anatomically, the AOB can be divided into the anterior and posterior AOB (aAOB and pAOB, respectively). Under light microscopy, the two regions exhibit similar layered structure and the glomerular layer (GL), mitral/tufted cell layer (MCL) and granule cell layer (GCL) are clearly distinguishable. However, the aAOB and pAOB differ significantly in their synaptic connections with the vomeronasal sensory neurons (VSNs). The aAOB receives axonal input from VSNs that have cell bodies residing in the apical region of the vomeronasal sensory epithelium. The pAOB, however, is targeted by VSNs that have cell bodies residing in the basal region of the epithelium (Halpern and Martínez-Marcos, 2003; Mucignat-Caretta, 2010). Apart from their cell body locations, these two populations of VSNs also differ in their expression of vomeronasal receptor families (V1R vs. V2R) and cell signaling proteins (Gαi2 vs. Gαo), suggesting they also differ in chemical response specificity (Jia and Halpern, 1996; Halpern et al., 1998; Kumar et al., 1999; Stowers and Kuo, 2015). Apically located VSNs are known to be sensitive to volatile pheromones and environmental signals, such as signals from predators and prey (Hagino-Yamagishi et al., 2001; Boschat et al., 2002; Del Punta et al., 2002; Keverne, 2004), while basally located VSNs preferentially respond to non-volatile semiochemicals, such as mouse major urinary proteins, major histocompatibility complex peptides and exocrine gland-secreting peptides (Krieger et al., 1999; Leinders-Zufall et al., 2004; Chamero et al., 2011). Additionally, the aAOB and pAOB contribute differently for AOB-mediated social and sexual behaviors (Dudley and Moss, 1999; Chamero et al., 2007, 2011; Tirindelli et al., 2009; Oboti et al., 2014; Pérez-Gómez et al., 2014). As such, the aAOB and pAOB process distinctive sensory inputs. Furthermore, there is evidence showing that the aAOB and pAOB send output signals to different regions of the brain (Martínez-Marcos and Halpern, 1999; Mohedano-Moriano et al., 2007). This indicates that the aAOB and pAOB may possess unique local neural networks or modulatory circuits to promote such segregated information processing and refinement.

Local interneurons play an important role in information processing of the AOB (Luo et al., 2003; Castro et al., 2007; Peretto and Paredes, 2014). While GABAergic, dopaminergic, and glutamatergic interneurons have been identified (Mugnaini et al., 1984; Takami et al., 1992; Hayashi et al., 1993; Yokosuka, 2012), the presence of cholinergic interneurons remains uncertain. Several attempts have been made to identify cholinergic neurons using antibodies against cholinergic markers choline acetyltransferase (ChAT), vesicular acetylcholine transporter (VAChT), and acetylcholinesterase (AChE), the enzyme that degrades ACh. Using these techniques, however, yielded inconsistent results and only a few studies show sporadic labeling of ChAT-expressing interneurons (Carson and Burd, 1980; Ojima et al., 1988; Ichikawa et al., 1997; Crespo et al., 1999). These previous investigations have demonstrated a limited capability of detecting interneurons using immunolabeling for cholinergic markers. As a result, the anatomical, morphological and physiological features of intrinsic cholinergic neurons, and their potential contribution to AOB information processing, remain largely unexplored.

Cholinergic activity in the MOB has been shown to influence sensory information processing via glomerular microcircuits and dendrodendritic synapses between granule cells and mitral/tufted cells (Nickell and Shipley, 1993; Pressler et al., 2007; Ghatpande and Gelperin, 2009; Rothermel et al., 2014; Bendahmane et al., 2016). Furthermore, boosting cholinergic activity improves discrimination of similar odorants (Linster et al., 2001; Chaudhury et al., 2009; Liu et al., 2015). More recent research showed that activation of muscarinic acetylcholine receptors (mAChRs) suppresses the activity of MOB mitral/tufted cells (Smith et al., 2015), while stimulation of the same receptors produces opposite effects in the AOB (Smith and Araneda, 2010). Similar to the MOB, the AOB receives heavy centrifugal projections from basal brain cholinergic neurons in the horizontal limb of the diagonal band of Broca (HLDB). Manipulation of this top-down cholinergic regulation critically influences mouse social behavior (Smith and Araneda, 2010). However, whether the centrifugal activity also modulates local cholinergic interneurons remains unknown.

Previously, we reported the presence of diverse populations of cholinergic interneurons in various layers of the MOB using antibodies against ChAT and VAChT, and a transgenic mouse line that allows excellent visualization of the cholinergic interneuron cell bodies (Krosnowski et al., 2012). In this line of transgenic mice, the expression of enhanced green fluorescent protein (eGFP) is driven by the endogenous ChAT transcriptional regulatory elements within a bacterial artificial chromosome (BAC; ChAT^(BAC)^-eGFP; Tallini et al., 2006). In this study, using the same transgenic mice and techniques, we detect and characterize a large quantity of putative cholinergic interneurons in the AOB, which were previously undocumented. The large number of diverse interneurons suggests potential physiological significance that has not yet been realized. Our characterization of these neurons further reveals that there are striking differences between aAOB and pAOB in their distribution, density and activation across various layers. Therefore, our results suggest that local cholinergic interneurons contribute significantly to the dichotomous information processing in the AOB.

## Materials and Methods

### Animals

Adult C57BL/6 background transgenic mice of both genders were used in this study. The original ChAT^(BAC)^-eGFP breeding pairs were kindly provided by Dr. M. I. Kotlikoff from Cornell University (Tallini et al., 2006). The expression of eGFP in cholinergic cells in this transgenic mice was characterized previously (Tallini et al., 2006; Ogura et al., 2007, 2011; Krasteva et al., 2011; Krosnowski et al., 2012). All animal care and procedures were approved by the Animal Care and Use Committee of University of Maryland, Baltimore County, MD, USA.

### Immunohistochemistry

#### Tissue Preparation

Our immunolabeling procedure has been described previously (Ogura et al., 2011; Krosnowski et al., 2012). Mice were deeply anesthetized with tribromoethanol (Avertin 250 μg/g body weight), perfusion-fixed with a phosphate buffered fixative containing 3% paraformaldehyde, 19 mM L-lysine monohydrochloride and 0.23% sodium m-periodate. The brain (including the OB) was harvested and post-fixed for 1.5 h before being transferred to 0.1 M phosphate buffered saline (PBS) with 25% sucrose overnight. The tissues were embedded and cut using a cryostat (Microm international, Walldorf, Germany) into free-floating 25 μm or 35 μm-thick sections that were either sagittal or parallel to the AOB outer vomeronasal nerve layer (VNL).

#### Immunohistochemistry

Brain sections containing AOB were rinsed in 0.1 M PBS 3× 10 min followed by 1.5 h incubation in PBS buffered blocking solution containing 2% normal donkey serum, 0.3% Triton X-100 and 1% bovine serum albumin. Sections were then immunoreacted for 48–72 h at 4°C with primary antibodies against each of the following proteins: GFP (1:3000; cat# ab13970, Abcam), Gαi2 (1:1000; cat# SC-13534, Santa Cruz), GABA (1:1000; cat# AB175, Sigma-Aldrich), VAChT (1:500; cat# V5387, Sigma), ChAT (1:250; cat# AB144P, Millipore, Billerica, MA, USA), calbindin D-28k (CB+; 1:2000; code No: 300, SWANT), tyrosine hydroxylase (TH; 1:3000; cat# 657012, Calbiochem), glutamate receptor type 2 and 3 (GluR2/3; 1:200; Clone ID:EP929Y, Epitomice, Inc.), c-Fos (1:1000; cat#PC38, Calbiochem) and vesicular glutamate transporter 2 (VGluT2; 1:2000; cat# AB5907, Millipore). For immnolabeling using the ChAT antibody, OB sections were pre-treated with either DAKO antigen retrieval solution (S2368) for 30 min in a 70°C water bath or with 0.1 M NaOH for 1–2 min, followed by three 30 s rinses of 0.1 M Na acetate and three 10 min washes with 0.1 M PBS. After incubation with primary antibodies, sections were washed and reacted with various secondary antibodies conjugated with either Alexa 555 or 647 (1:400; Invitrogen) or Alexa 488 donkey anti-Chicken secondary antibody (1:400; Jackson ImmunoResearch) for 1 h at room temperature. Sections were mounted on slides with Fluoromount-G containing DAPI, which stains nuclei (Southern Biotech). In control experiments, primary antibodies were omitted, which resulted in negative labeling.

#### Image Acquisition

An Olympus BX 41 epi-fluorescence compound microscope, equipped with a Retiga 4000R camera (QImaging, Surrey, BC, Canada) and Image-Pro Plus 6.2 (Media cybernetics, Bethesda, MD, USA), was used to acquire low magnification images. High magnification images of immunolabeled sections were taken using an Olympus BX 61 epifluorescence microscope equipped with a spinning disc confocal unit and Slidebook 4.0 software (3i, Denver, CO, USA).

### Cell Counting and Volume Measurement for Cell Density Estimation

Every third bulbar section containing AOB was cut sequentially from either the right or left OBs of individual mice. These sections were immunolabeled with antibodies against GFP and Gαi2, which marks the aAOB. Additionally, AOB sections were also immunolabeled with antibodies for particular interneuron cell types, such as TH-expressing (TH+) or CB+ interneurons. Epi-fluorescence images of the AOB were taken using a 10x lens and a Retiga 4000R camera. Cell counts for cholinergic (GFP+) interneurons were conducted manually on each AOB section. Boundaries for each layer were estimated based on anatomical landmarks, GFP and Gαi2 antibody labeling, DAPI and characteristics of the lateral olfactory tract (LOT). The midline separating the aAOB and pAOB was determined by extending the boundary between the anterior and posterior GL, evident from labeling with Gαi2 through the remaining layers, which was roughly perpendicularly to the AOB outer surface. To estimate the total volume of the aAOB or pAOB, all AOB sections from each individual bulb were processed. The volume of AOB region was calculated by multiplying the thickness of each section with the area occupied by each region, measured in NIH ImageJ. The volume for each tissue section was summed to estimate the total volume of each AOB region. To estimate of the number of GFP+ neurons per layer, the number of cells from every third bulbar section were counted and multiplied by three. The number of counted cells was divided by the total volume of each layer to determine the cell density.

### Quantification of Cholinergic Nerve Processes Using Line Intensity Profile Analysis

Our method of counting nerve fibers, which is based on line intensity scan analysis using an image processing filter and peak detection algorithm, has been described in detail in our previous publications (Krosnowski et al., 2012; Sathyanesan et al., 2012). For this analysis, we used immunofluorescence images of VAChT immunoreactivity (VAChT-ir) taken using a 4x lens from sequential OB sections (210 μm apart). The images were first processed for Hessian-based feature extraction before the line intensity scan analysis, which was performed by drawing segmented lines through the GL, MCL, located between the MCL and GCL, and GCL individually, parallel to the outer edge of each layer, using NIH ImageJ software. Fluorescence intensity values along the line drawn on the image were extracted as a line intensity profile. Background fluorescence intensity was set to the average intensity of a line intensity scan from the VNL, where there is no detectable VAChT-ir. Intensity peaks, which represent individual nerve fibers, were detected using a peak-detection algorithm from the MATLAB Bioinformatics toolbox, “mspeaks”, and the total number of peaks was then divided by the length of each individually drawn segmented line to yield the average number of peaks per 1 μm long line. In addition to the parallel scan, we also performed additional line scans perpendicular to the curvature of the parallel line scan for each layer within the aAOB and pAOB. This was done to account for the fibers running parallel to the layer. The number of peaks obtained through the parallel scans and the number of peaks obtained through the perpendicular scans were multiplied to yield total number of peaks per unit area of the section. For estimation of volumetric density (fibers/(100 μm)^3^), the peaks per unit area was divided by section thickness and multiplied by 10^6^. Obtained volumetric density is adjusted to account for the random orientation of fibers as well as optical signal loss or attenuation in the section (divided by cos 45° and 1/1.6, respectively; Sathyanesan et al., 2012).

### Complex Odor Exposure and c-Fos Immunolabeling to Monitor AOB Neuronal Activation

For stimulation of the VNO with complex odors, two sources of stimuli were used. Either a male aggressor (3–7 months old) taken directly from our mating cages from our animal facility, or soiled bedding freshly collected from a cage housing a mating pair (2–7 months old; unchanged for at least 2 days) was used. The procedures used for odor stimulation and c-Fos immunolabeling were described in our previous publications (Lin et al., 2004, 2007), with the following modifications. Individual sexually naïve adult male mice (2.5–7 months old) were transferred to a clean cage and singly housed overnight. The next day, either a sexually experienced male aggressor mouse or soiled bedding from a mating pair cage was gently introduced in the home cages of experimental mice. The experimental mouse was allowed to move freely and make contact with the stimuli. The male aggressor remained in the cage for 30 min. Ninety minutes following the start of exposure, mice were euthanized, OB tissue was obtained and every third section was processed for immunolabeling using antibodies against GFP, c-Fos and Gαi2. Control mice were not exposed to stimuli and were processed identically. Manual cell counting was performed to determine the ratio of cells positive for both c-Fos and GFP against the total number of GFP^+^ cells. Counts were conducted in both the anterior and posterior MCL only.

### Data Analysis

Cell counts are reported as averages of data from three different mice ± the standard error of the mean (SEM). Student’s *t*-tests were used to compare number of GFP+, CB+, and FOS+ cells as well as densities of VAChT immunolabeled nerve fibers in aAOB and pAOB regions. *p* < 0.05 was considered statistically significant.

## Results

### Dichotomous Distribution of Putative Cholinergic Neurons in the AOB

**Figure 1:**
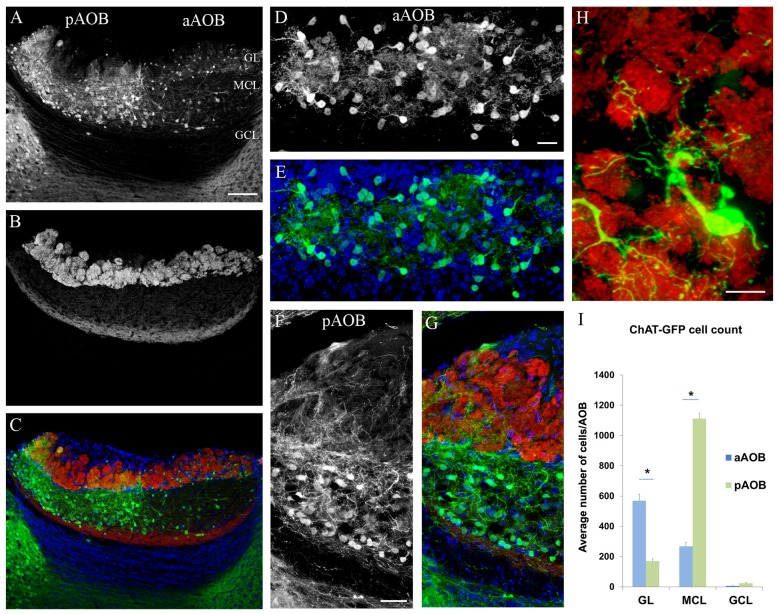
**Dichotomous distribution of cholinergic neurons in the accessory olfactory bulb (AOB). (A–C)** Confocal images of the AOB taken from a sagittal OB section of choline acetyltransferase (ChAT)^(BAC)^-enhanced green fluorescent protein (eGFP) mouse, showing the distribution of GFP+ cholinergic neurons. **(A)** GFP immunoreactivity (ir). Note that GFP+ neurons are densely populated in the aAOB glomerular layer (aGL) and pAOB mitral/tufted cell layer (pMCL) contrasting to the sparse occurrence in the pGL and aAOB MCL (aMCL). **(B)** Vesicular glutamate transporter 2 (VgluT2-ir) in the GL layer. **(C)** Overlay of GFP-ir (**A**; green) and VgluT2-ir (**B**; red). GL, glomerular layer. MCL, mitral cell layer. GCL, granule cell layer. aAOB and pAOB, anterior and posterior AOB, respectively. The section was counterstained with DAPI (blue). **(D)** GFP+ juxtaglomerular interneurons in the aGL. **(E)** Overlay of GFP-ir (**D**; green) and DAPI (blue). **(F)** GFP+ neurons in the pMCL. Note GFP+ nerve processes enter the pGL from the pMCL. **(G)** Overlay of GFP-ir (**F**; in green), VgluT2-ir (red) and DAPI stain (blue). **(H)** A GFP+ interneuron in the aGL sends nerve processes to multiple glomeruli visualized with VgluT2-ir. **(I)** Plot of average number of GFP+ neurons in GL, MCL and GCL of aAOB and pAOB per AOB (Mean ± SEM). The differences in the number of GFP+ neurons in the GL and MCL between the aAOB and pAOB are statistically significant (Student’s *t*-test, *n* = 3 mice. *Marks statistical significance *p* < 0.05). Scale: **(A–C)** 100 μm. **(D–G)** 20 μm. **(H)** 10 μm.

The mouse AOB is commonly divided into four layers (from surface to center): the nerve layer made up of VSN axons (VNL), GL, MCL and GCL (Halpern and Martínez-Marcos, 2003; Yokosuka, 2012; Smith et al., 2015; Gorin et al., 2016). We first investigated whether cholinergic interneurons of the AOB are present and exhibit any dichotomous distribution patterns in the anatomically and functionally segregated aAOB and pAOB. In order to discern the different layers of the AOB, we immunolabeled sagittal OB sections from ChAT^(BAC)^-eGFP mice with an antibody against VgluT2, which labels the VSN axon terminals in glomeruli of the GL. We also used an anti-GFP antibody to intensify GFP signal in both cell bodies and nerve fibers. We found GFP+ neurons located predominantly in the GL and MCL of the AOB (Figure 1A: image of GFP immunoreactivity (ir) showing GFP+ neurons; Figure 1B: image of VgluT2-ir; Figure 1C: overlay of **1A,B** and DAPI counterstaining). There were striking differences in the GFP+ interneuron cell populations between the aAOB and pAOB. Significantly more GFP+ cells were present in the GL of the aAOB (aGL) than in the GL of the pAOB (pGL; Figure 1D: GFP+ interneurons in the aGL; Figure 1E: overlay with DAPI image). These GFP+ juxtaglomerular interneurons commonly sent processes to several adjacent glomeruli labeled with the VgluT2 antibody (Figure 1H: a GFP+ interneuron innervates several VgluT2-positive glomeruli). In contrast, in the MCL, GFP+ neurons were present in significantly higher density in the pAOB MCL (pMCL) than in the aAOB MCL (aMCL; Figure 1F, GFP-ir image taken from the pMCL, Figure 1G, overlay with VgluT2-ir and DAPI images). Within the pMCL, the GFP+ cells were not uniformly distributed; instead they were often more concentrated in its border region. In the aMCL, only a few GFP+ neurons were found. These distinctive patterns of cholinergic cell distribution in the AOB were highly consistent between individual animals and to our knowledge, have not been reported. Therefore, our results present the first evidence showing that local putative cholinergic interneurons may participate in differential information processing within the AOB.

### Quantitative Analysis of the AOB Putative Cholinergic Interneurons

We next quantified the difference in the number of GFP+ neurons between the aAOB and pAOB. The different layers and regions of the AOB were distinguished by immunolabeling using antibodies against the Gαi2 and VgluT2. We counted GFP+ neurons from every third AOB sagittal section for each individual bulb and measured the total volume of each layer within the aAOB or pAOB to estimate the total number of GFP+ cells per layer. The results are shown in Figure 1I. On average, there were approximately 570 GFP^+^ cells in the aGL, 267 GFP^+^ cells in the aMCL, and 7 GFP^+^ cells in the aGCL per aAOB. In the pAOB, we found 170 GFP+ cells in the pGL, 1113 GFP+ cells in the pMCL, and 23 GFP+ cells in the GCL per pAOB. Statistical data analysis indicated highly significant differences in the GFP+ neuron count between the aAOB and pAOB in both the GL and MCL (Student’s *t*-test, *p* < 0.05, *n* = 3 mice). Therefore, our data indicate distinct cholinergic neuron populations that may selectively influence sensory information processing in these regions.

### Immunoreactivity of Cholinergic Marker Vesicular Acetylcholine Transporter (VAChT) and ChAT

**Figure 2:**
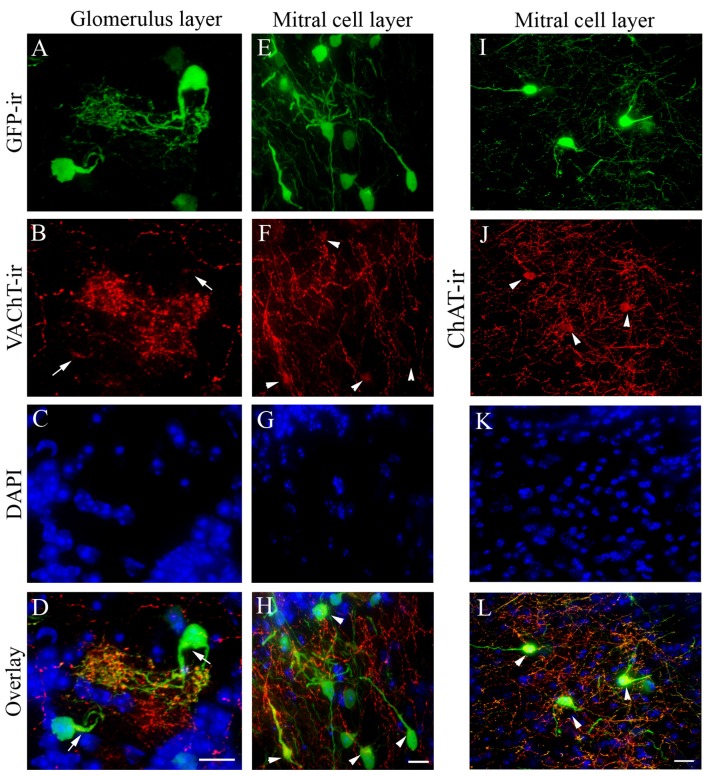
**Vesicular acetylcholine transporter (VAChT) immunoreactivity in cholinergic nerve processes in the AOB.** Sagittal AOB sections of ChAT^(BAC)^-eGFP mice were labeled with antibodies against GFP and VAChT. **(A)** GFP+ juxtaglomerular interneurons in the aGL. Note extensive nerve processes from a GFP+ neuron. **(B)** VAChT-ir. Strong VAChT-ir is present in the distal nerve processes, while in the cell body and proximal processes the labels is very weak and only present in certain regions pointed by arrows. **(C)** DAPI counterstain, which roughly outlines individual glomeruli. **(D)** Overlay of GFP-ir (**A**; green), VAChT-ir (**B**; red) and DAPI (**C**; blue). **(E–H)** Confocal images taken from the middle of the MCL. **(E)** GFP-ir. **(F)** VAChT-ir. Many nerve processes are labeled. Arrowheads point to some cell bodies of GFP+ neurons that show relatively weak VAChT-ir. **(G)** DAPI counterstain. **(H)** Overlay of GFP-ir (**E**; green), VAChT-ir (**F**; red) and DAPI (**G**; blue). **(I–L)** Confocal images taken from the aMCL of a section immunoreacted with antibodies against ChAT and GFP. The section was cut parallel to the AOB surface. **(I)** GFP+ neurons in the aMCL, **(I)** GFP-ir **(J)** ChAT-ir, **(K)** DAPI **(L)** Overlay of GFP-ir (**I**; green), ChAT-ir (**J**; red), DAPI (**K**; blue). Scale: **(A–D)** 10 μm. **(E–L)** 20 μm.

We next examined the expression of VAChT and ChAT in GFP+ cells of the AOB sections using immunolabeling. The VAChT marker is of interest because it transports ACh into vesicles at presynaptic terminals. Similar to our previous results in the MOB (Krosnowski et al., 2012), the VAChT antibody labeled numerous nerve processes in the AOB. In the aGL, VAChT immunolabeling was observed mostly in the nerve processes within the glomeruli, which roughly were outlined by the DAPI counterstaining of the nuclei of periglomerular cells (Figure 2A: GFP+ neurons in the aGL; Figure 2B: VAChT-ir; Figure 2C: DAPI; Figure 2D: Overlay). We found that VAChT-ir in GFP+ cell bodies was weak and visible only in small regions (pointed by arrows). This was difficult to locate without using GFP to visualize the cell body. In the MCL, the VAChT antibody labeled numerous fine processes, which mostly might be originated from centrifugal projection (Figure 2E: GFP+ neurons in the middle of MCL, Figure 2F: VAChT-ir; Figure 2G: DAPI, Figure 2H: overlay). VAChT-ir in proximal regions of the long processes of GFP+ neurons in the MCL was often limited to only small segments (pointed by arrow heads). Some GFP+ cells did not show visible VAChT labeling. Similar weak staining for ChAT was also found in the cell bodies although some GFP+ cells in the aMCL showed a better signal (Figure 2I, image of GFP+ cells in the aMCL taken from a section cut parallel to the surface of the AOB, Figure 2J: ChAT-ir, Figure 2K: DAPI, Figure 2L: overlay). These results, confirmed the cholinergic status of many GFP+ cells.

### Unique Distribution and Nerve Fiber Orientation of Putative MCL Cholinergic Interneurons

**Figure 3:**
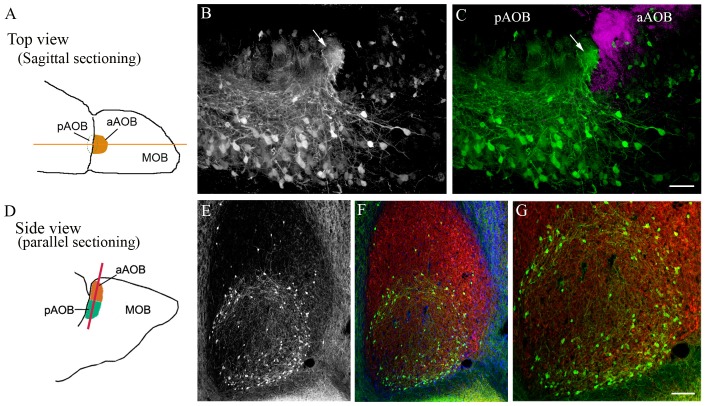
**Unique distribution pattern of cholinergic neurons in the pMCL of the AOB. (A)** A schematic drawing of an AOB in a left OB (top view). The orange line denotes the sagittal cutting plane. **(B)** GFP-ir image taken from a sagittal AOB section. GFP+ neurons are densely populated in the pMCL. **(C)** Overlay of GFP-ir (**B**; green and Gαi2-ir images; magenta). The aAOB receives vomeronasal sensory nerve positive for Gαi2-ir. A significant higher number of GFP+ neurons is found in the pMCL than in the aMCL. GFP+ nerve fibers from the MCL enter and form a plexus in middle of the GL negative for Gαi2-ir, (pointed by an arrow). **(D)** A schematic drawing of the AOB (side view). The orange line denotes the cutting plane parallel to the AOB surface. **(E–G)** Images taken from a parallel-cut section through the MCL. **(E)** GFP-ir. Most GFP+ neurons are located near the border region within the pMCL. Only a few GFP+ neurons are present in the aMCL. **(F)** Overlay of GFP-ir (**E**; green), DAPI (blue) and glutamate receptor type 2 and 3 (GluR2/3)-ir (red), which labels mitral/tufted cells and their processes in both the aMCL and pMCL. **(G)** A higher magnification image showing GFP+ neurons and their nerve processes in the outer region of the pMCL. Scale: **(B,C)** 50 μm. **(E–G)** 100 μm.

As viewed from AOB sagittal sections, neurons in the MCL send processes into the glomeruli, especially those positioned near the boundary within the pGL (Figure 3A: schematic drawing of an OB viewed from the top, the yellow line indicates sagittal sectioning. Figure 3B: GFP+ neurons; Figure 3C: overlay with Gαi2-ir; magenta, see also Figures 1A,C). In comparison to the GFP+ cells of the GL, pMCL GFP+ neurons possess relatively larger cell bodies and much longer processes. Because of the high cell density, the morphological features of the GFP+ neurons in the pMCL are difficult to discern. Thus, we could not determine whether the cells located near the border region differ from those located in the center region in their fiber orientation. Interestingly, some GFP+ neurons in the aMCL protrude two major processes from two opposite ends, and one of these processes occasionally were seen to pass the midline and enter the pMCL (Figures 3B,C). Some GFP+ neurons in the aMCL also sent processes to the strong GFP+ glomeruli in the middle of the GL.

We were intrigued by the dichotomous distribution of GFP^+^ neurons between the aAOB and pAOB and the nerve fiber orientation of the pMCL GFP+ neurons, and decided to section the AOB parallel to its outer surface (Figure 3D, schematic drawing of an OB with the orange line indicating parallel sectioning). The MCL was visualized by GluR2/3-ir, which labels glutamatergic mitral/tufted cell bodies and processes (Figure 3F: GluR2/3-ir in red; overlay with GFP-ir image). With this cutting plane, the striking patterns of the cholinergic cell distribution in the MCL are clearly evident (Figure 3E: GFP+ in the MCL). GFP+ neurons in the pMCL formed a circular ring near border region within the pAOB, and more GFP+ cells were located in the outer region than in the center (Figures 3E–G, with enlarged images in **3G**). Nerve fibers emanating from the GFP+ neurons in the pMCL were clearly visible and some apparently ran along the outer region within the pMCL. This innervation pattern was not observed in the aMCL, suggesting distinctive cholinergic modulation between the two regions.

### Characterization of Glomeruli that Receive High Density of GFP+ Nerve Fibers within the AOB

**Figure 4:**
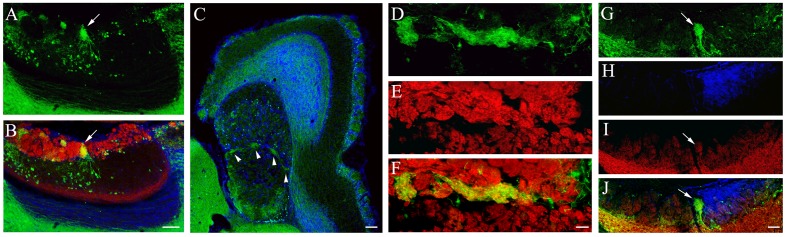
**Characterization of glomeruli in the AOB that receive strong cholinergic innervation. (A,B)** Images were taken from a sagittal OB section. **(A)** GFP-ir. **(B)** overlay of GFP-ir (green) VgluT2-ir (red) and DAPI (blue). A large GFP+ glomerulus in the middle of the GL receives dense cholinergic nerve innervation (pointed by an arrow). **(C–F)** Images taken from parallel-cut sections through the GL of the AOB. **(C)** A low magnification image of GFP-ir (green) and DAPI (blue). Several glomeruli at the middle of the AOB are visible (pointed by arrow heads). Note somewhat diffuse yet strong GFP-ir in the caudal region of the pGL, indicating cholinergic innervation to the pGL. **(D)** GFP-ir. **(E)** VgluT2-ir. **(F)** Overlay of GFP-ir (**D**; green) and VgluT2-ir (**E**; red). Regions innervated by GFP+ nerve fibers are positive for VgluT2-ir. **(G–J)** Images taken from a sagittal section immunoreacted with antibodies against GFP, Gαi2 and GluR2/3. **(G)** GFP-ir. **(H)** Gαi2-ir. **(I)** GluR2/3-ir. **(J)** Overlay of GFP-ir (**G**; green), Gαi2-ir (**H**; blue) and GluR2/3-ir (**I**; red). The glomerulus (pointed by an arrow) that receives strong cholinergic (GFP+) innervation is also positive for GluR2/3-ir, indicating innervation from mitral/tufted cells. Note that the glomerulus is negative for Gαi2-ir, indicating that it does not belong to the aAOB. Scale: **(A–C)** 100 μm. **(D–F)** 20 μm. **(G–J)** 50 μm.

In sagittal sections of the AOB, we consistently found a relatively large glomerulus that resided in the middle of the GL that exhibited a strong GFP signal. The GFP+ nerve fiber plexus was primarily composed of local GFP+ neurons located in the MCL (Figure 4A: GFP-ir image of AOB, an arrow points the large middle glomerulus; Figure 4B: overlay with VgluT2-ir image). In some sections, we also sometimes observed other small glomeruli with strong GFP signal in the pGL. We further examined the distribution of these glomeruli in AOB sections cut parallel to the surface of the VNL. There were several strong GFP+ plexuses present along the boundary between the aAOB and pAOB (Figure 4C pointed by arrowheads). We confirmed their glomerular status by immunolabeling the AOB sections with the anti-VgluT2 antibody (Figure 4D: GFP-ir; Figure 4E: VgluT2-ir; Figure 4F: overlay). Because glomeruli in the AOB do not have a clear boundary, it was difficult to discern the exact number of glomeruli that exhibited strong GFP. However, this unique pattern of GFP+ glomeruli was very consistent between the left and right bulbs and among individual mice cutting with the parallel plane (*n* = 4 mice). We next determined whether these strong GFP+ glomeruli also receive glutamatergic innervation from mitral/tufted cells by immunolabeling sagittal AOB sections with antibodies against GluR2/3 and Gαi2. The anti-GluR2/3 antibody strongly labeled mitral/tufted cells in the MCL. Although the GluR2/3-ir in glomeruli is relatively weaker, it is clearly visible in the glomerulus with strong GFP+ (Figure 4G: GFP-ir; Figure 4H: Gαi2-ir; Figure 4I: GluR2/3-ir; Figure 4J: overlay. An arrow points the GFP+ glomerulus), indicating that sensory information from the strong GFP+ glomeruli is also relayed to mitral/tufted output neurons. Therefore, our data identified a unique small population of glomeruli within the AOB that receive strong innervation from putative cholinergic neurons in the MCL.

### The GFP+ Putative Cholinergic Interneurons of the AOB Are neither GABAergic nor Dopaminergic

**Figure 5:**
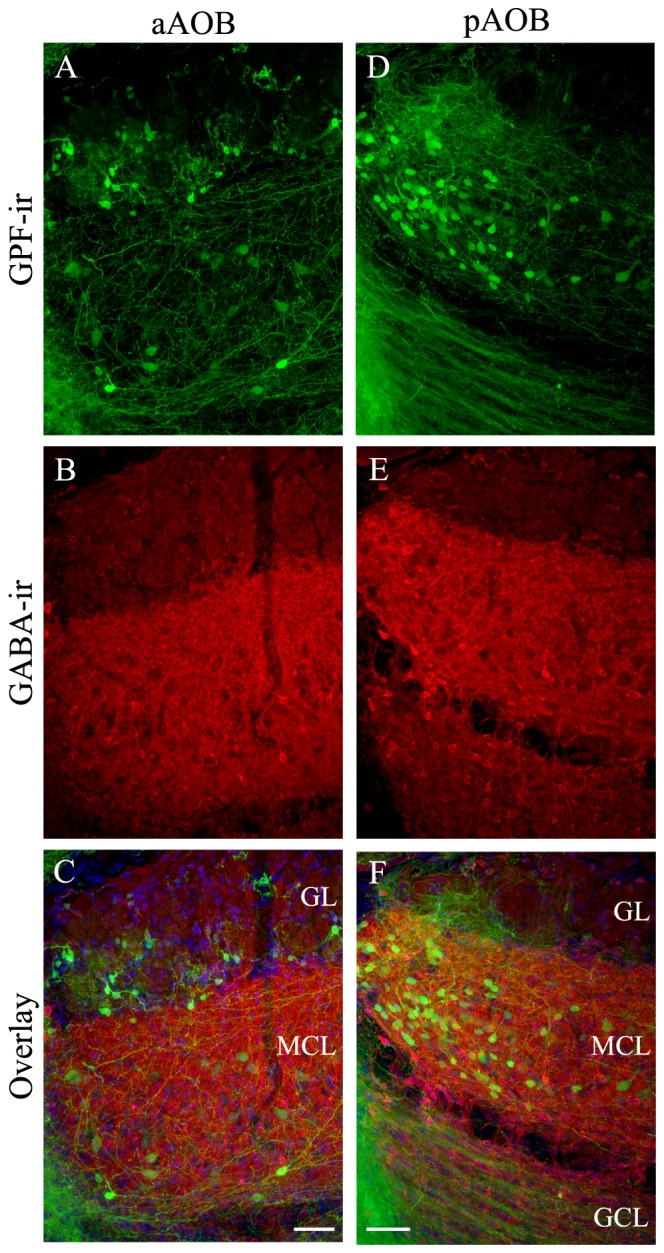
**Cholinergic neurons in the AOB are not GABAergic. (A–F)** are confocal images of the aAOB and pAOB, respectively. These images were taken from sagittal AOB sections immunoreacted with antibodies against GFP and GABA. **(A,D)** GFP-ir. **(B,E)** GABA-ir. Intense GABA-ir is present in the MCL and GL (both cell bodies and processes). **(C,F)** Overlays of GFP-ir (**A,D**; green) GABA-ir (red; **B,E**) and DAPI (blue) respectively. In all layers, GFP+ neurons are negative for GABA-ir, indicating cholinergic and GABAergic neurons belong to separate populations. Scale: 50 μm.

**Figure 6:**
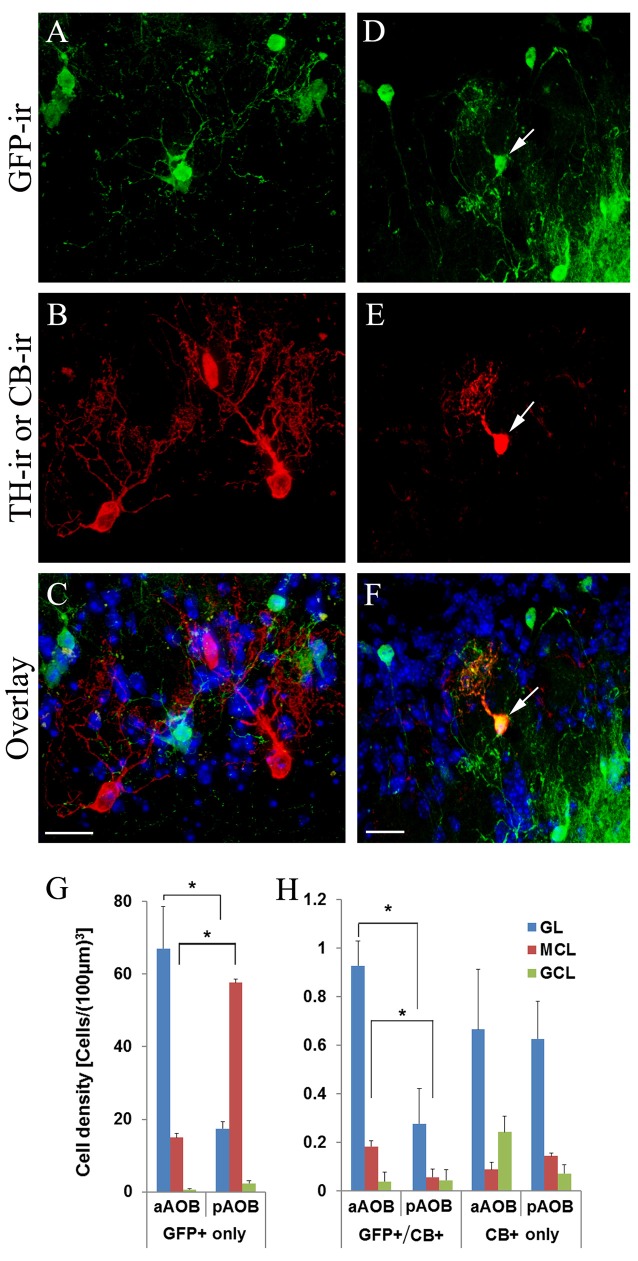
**Cholinergic interneurons are distinct from dopaminergic interneurons but partially overlap with interneurons expressing calbindin D-28k. (A–C)** Images from the aGL in a sagittal AOB section immunoreacted with antibodies against GFP and tyrosine hydroxylase (TH). **(A)** GFP-ir. **(B)** TH-ir. **(C)** Overlay of GFP-ir (**A**; green), TH-ir (**B**; red) and DAPI (blue). There is no overlap between GFP+ and TH-ir positive interneurons. **(D–F)** Images of a sagittal AOB section immunoreacted with antibodies against GFP and calbindin D-28k in the pGL. **(D)** GFP-ir. **(E)** CB-ir. **(F)** Overlay of GFP-ir (**D**; green), (**E**; red) and DAPI (blue). An arrow points a GFP+ interneuron positive for CB-ir. **(G)** Density of GFP-expressing cells solely. **(H)** Density of GFP and CB co-expressing cells and CB expressing cells solely (Mean ± SEM). The number of CB+ cells is significantly lower than that of GFP+ cells. A subset of CB+ interneurons also is GFP+, suggesting partial overlap between the two populations. There are significantly more GFP+/CB+ cells in the aGL and aMCL than in the pGL and pMCL, respectively (Student’s *t*-test, *n* = 3 mice. *Indicates statistical significance *p* < 0.05). Scale: 20 μm.

Next, we determined if the putative cholinergic interneuron populations are distinct from other known interneurons present in the AOB using immunolabeling of various cell markers. We found that the antibody against GABA, the neurotransmitter used in GABAergic interneurons, labels numerous interneurons in all three layers in both the aAOB (Figures 5A–C) and pAOB (Figures 5D,E), respectively (Figures 5A,D: GFP-ir; Figures 5B,E: GABA-ir; Figures 5C,F: overlay). None of the GFP+ neurons were positive for GABA-ir. Therefore the cholinergic and GABAergic neurons belong to distinct populations. We also labeled AOB sections with an antibody against TH, which serves as the rate limiting enzyme for dopamine synthesis in dopaminergic interneurons. There was no co-localization of GFP and TH-ir (Figures 6A–C images taken from the aGL. Figure 6A: GFP-ir; Figure 6B: TH-ir; Figure 6C: overlay). Therefore, the GFP+ cells are not dopaminergic.

### Some GFP+ Interneurons Are Positively Labeled with Antibodies against Calbindin D-28k (CB)

We previously have shown that a subset of cholinergic interneurons in the GL of the MOB express CB (CB+; Krosnowski et al., 2012). Therefore, we investigated whether there could be an overlap between GFP+ neurons and CB+ neurons in the AOB. We found that some of the GFP+ interneurons were positively labeled with the antibody against CB (Figure 6D: GFP; Figure 6E: CB-ir; Figure 6F: overlay). We performed the cell count and calculated the density values of neurons that were either solely GFP+ (Figure 6G), or solely CB+ as well as those that were both GFP+ and CB+ in different layers of the aAOB and pAOB (Figure 6H). There were significantly more GFP+ cells than CB+ cells in the GL and MCL (Student’s *t*-test, *p* < 0.05, *n* = 3 mice). Interestingly, there were more CB+ cells in the aAOB than the pAOB, most of which were found in the aGL and also GFP+. This result indicates that the GFP+ interneurons in the AOB share a cytochemical feature with cholinergic interneurons in the MOB.

### Most of the GFP+ Putative Cholinergic Interneurons of the MCL Are Not Glutamatergic

**Figure 7:**
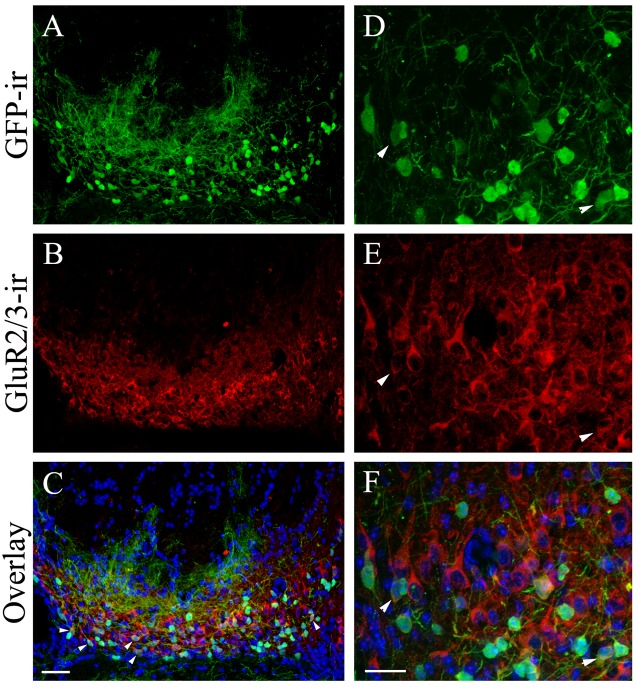
**The majority of the GFP+ neurons in the pMCL are not glutamatergic.** Confocal images of caudal pMCL taken from AOB sections cut parallel to the AOB surface. The sections were immunoreacted with antibodies against GFP and GluR2/3. **(A–F)** Images of low and relatively higher magnification, respectively. **(A,D)** GFP-ir. **(B,E)** GluR2/3-ir. **(C,F)** Overlays of GFP-ir (**A,D**; green), GluR2/3-ir (red; **B,E**, respectively) and DAPI (blue). Most of the GFP+ neurons are negative for GluR-ir. However, a subset of pMCL neurons is both GFP and GluR2/3-ir positive, indicating a small overlap of cholinergic and glutamatergic neuron populations in the pMCL. Scale: 20 μm.

The MCL houses the cell bodies of mitral/tufted cells. Because of the large number of GFP+ cells in the MCL and their innervation of glomeruli, we performed double immunolabeling using antibodies against GFP and the glutamatergic cell marker GluR 2/3, which labels mitral/tufted cells. The result is shown in Figures 7A–F, which are low and higher magnification images, respectively, taken from the caudal region of the pMCL from sections cut parallel to the outer surface of the VNL. The anti-GluR2/3 antibody labeled numerous mitral/tufted cells in the MCL. Most of the GFP+ cholinergic cells were negative for GluR 2/3-ir, suggesting they are not glutamatergic. The GFP+ cells also showed slightly smaller cell bodies with thinner processes compared to GluR 2/3-ir positive mitral/tufted cells (Figures 7A,D: GFP; Figures 7B,E: GluR2/3-ir; Figures 7C,F: overlay). Surprisingly, we also found that some GFP+ cells in the pAOB that were positively labeled by the anti-GluR 2/3 antibody (pointed by arrowheads in Figures 7D–F). These data suggest that a small subset of GFP+ cells in the pMCL might be both cholinergic and glutamatergic.

### Quantitative Analysis of Cholinergic Nerve Fiber Density in Various Layers of the AOB

**Figure 8:**
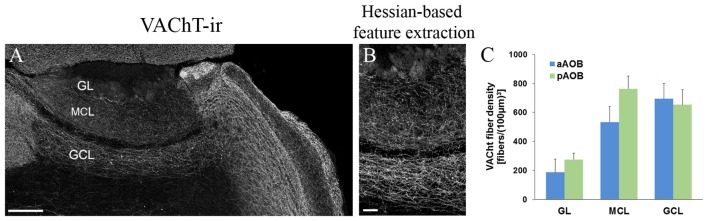
**Quantitative analysis of cholinergic nerve fiber density in various layers of the aAOB and pAOB. (A)** A low-magnification image of the AOB taken from a sagittal section. VAChT-ir positive nerve fibers are present in the GL, MCL and GCL. **(B)** Image of the enlarged middle AOB region that has been processed using Hessian-based feature extraction for automatic fiber count (for detailed method, see Krosnowski et al., 2012 and Sathyanesan et al., 2012). **(C)** Plot of VAChT-ir fiber density (Mean ± SEM, *n* = 3 mice). A higher density of VAChT-ir positive fibers is found in MCL and GCL than in the GL, with the fiber density in the pMCL trending toward being the highest. Scale: **(A)** 200 μm **(B)** 50 μm.

We have previously quantified cholinergic nerve fibers in the MOB (Krosnowski et al., 2012) using our published individual nerve fiber counting method (Sathyanesan et al., 2012). Recently, Smith et al. (2015) reported the difference in fiber density between the MOB and AOB by determining overall fluorescence intensity of the cholinergic (GFP+) fibers using a different transgenic mouse line. However, the differences between the aAOB and pAOB were not reported. We performed a cholinergic nerve fiber count on various layers of the aAOB and pAOB. In order to obtain accurate nerve fiber count from large area, we used AOB sections immunolabeled with the antibody against VAChT because it primary labels nerve fibers (Krosnowski et al., 2012; Sathyanesan et al., 2012; Figure 8A). The images of VAChT-ir were also processed for Hessian-based feature extraction to enhance the signal to noise ratio (Figure 8B, for detailed method, see Sathyanesan et al., 2012). We found the majority of VAChT-ir positive cholinergic fibers were located in the MCL and GCL of the AOB (Figure 8C). The highest fibers density was found in the pMCL, although there was no statistical difference from the density value in the aMCL (Student’s *t*-test, *n* = 3 mice, *p* = 0.085). The density values in the aGCL and pGCL were similar. The presence of these extensive cholinergic fiber networks in the AOB indicates the potential important role of cholinergic modulation in all layers.

### Activation of GFP+ Putative Cholinergic Interneurons by Vomeronasal Sensory Inputs

**Figure 9:**
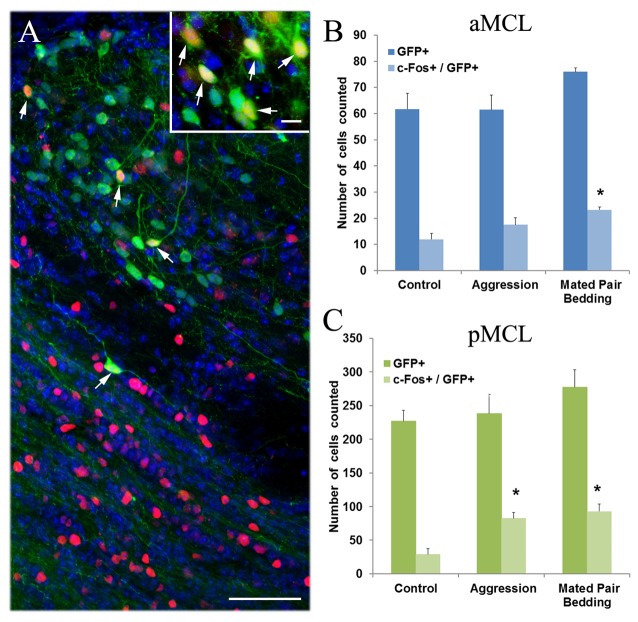
**Semiochemical-induced c-Fos protein expression in cholinergic interneurons of the AOB. (A)** An image of pAOB taken from a sagittal AOB section from a mouse exposed to a male aggressor and immunoreacted with antibodies against both GFP and c-Fos. Many c-Fos-expressing interneurons (red) are found in pGCL and some in pMCL. Arrows point to GFP+ neurons (green) that express c-Fos. DAPI counter stain (blue) Inset: an image of GFP+ cells that expresses c-Fos from another section. **(B,C)** Plots of counts for GFP+ cells and cells that show both GFP and c-Fos-ir in the aMCL and pMCL, respectively. Values presented are an average number of the total cells counted from every third serial section (mean ± SEM). In the aMCL, the number of c-fos+/GFP+ cells increases significantly after exposure to soiled bedding from a mating pair cage while in the pMCL, exposure to both a male aggressor and soiled bedding increases the number of c-Fos-expressing cells significantly when compared to the unstimulated control (Student’s *t*-test, *p* < 0.05, *n* = 4 mice. *Indicates statistical significance). Scale: **(A)** 50 μm. inset: 10 μm.

To determine whether the GFP+ neurons in the AOB can be activated by sensory inputs, we exposed naïve male mice to either a sexually experienced male aggressor mouse or soiled bedding collected from mating cages. These complex stimuli are known to induce neuronal activation in the AOB (Kumar et al., 1999; Matsuoka et al., 1999; Portillo and Paredes, 2004) which can be monitored by immunolabeling of c-Fos protein (Figure 9A; representative c-Fos expressing GFP+ neurons is pointed by arrows. Inset: c-Fos-expressing GFP+ cells from another section). We manually counted the number of GFP+ cells activated (Fos+) in the MCL of the aAOB and pAOB in sections immunolabeled with antibodies against Gαi2 and c-Fos protein. In the aMCL, we observed a significant increase in the number of c-Fos+/GFP+ cells after stimulation with soiled bedding from a mating pair (Figure 9B; plot of the numbers of GFP+ cell alone and c-Fos-positive GFP+ cell counted in the aMCL. *n* = 4 mice, Student’s *t-test*, *p* < 0.05). The number of c-Fos-positive GFP+ cells was also increased when stimulated with a male aggressor in comparison to the unstimulated control, but the increase was not significantly different. In the pMCL, both stimuli increased the number of c-Fos+/GFP+ cells significantly when compared to unstimulated control animals (Figure 9C, plot of GFP+ cell and c-Fos-positive GFP+ cell counts in the pMCL. *n* = 4 mice, Student’s *t*-test, *p* < 0.05). These results indicate that GFP+ putative cholinergic neurons in the AOB participate in processing social and sexual related stimuli.

## Discussion

We have provided evidence of local putative cholinergic interneurons in the AOB. We showed that these cells are located primarily in the GL and MCL and that there are striking differences in the density and distribution between the aAOB and pAOB. We also uncovered unique patterns of putative cholinergic neurons in the pMCL and identified a specific set of glomeruli in the middle of the GL that receive strong local cholinergic innervation. We confirmed that many of these putative cholinergic AOB neurons express the cholinergic markers ChAT and VAChT. Additionally, we determined that these putative cholinergic neurons are neither GABAergic nor dopaminergic and most of them are distinct from the glutamatergic mitral/tufted cells. Furthermore, we showed that exposure to either a sexually experienced male aggressor or soiled bedding from a mating cage leads to activation of some putative cholinergic cells in the AOB. Taken together, our results revealed novel populations of putative cholinergic neurons in the AOB. The significant differences in their distribution and cell density between the aAOB and pAOB imply a potentially important role in dichotomous information processing between the two regions.

The AOB neurons receive and process dichotomous sensory inputs from the VNO evoked by conspecific and allospecific cues present in bodily secretions, such as urine and scent markings. These complex odor blends vary substantially among individual mice and are indicative of the producer’s identity, social and sexual statuses, and environment (Halpern and Martínez-Marcos, 2003; He et al., 2008; Ben-Shaul et al., 2010; Mucignat-Caretta, 2010; Kaur et al., 2014; Peretto and Paredes, 2014; Wyatt, 2014; Fu et al., 2015). Adding to this complexity, VSN activity patterns in response to these stimuli are also dynamic and can differ significantly depending on the receiver’s reproductive stages and sexual experience, suggesting interpretation of these pheromonal stimuli also varies among individuals (Segovia and Guillamón, 1993; Rodriguez and Boehm, 2009; Dey et al., 2015; Stowers and Kuo, 2015; Stowers and Liberles, 2016; Xu et al., 2016). This evidence points to specialized information processing in the AOB that is highly plastic and regulated by both global and local networks based on the overall physiological state and special conditions of social and sexual status of animals.

Among various top-down regulatory systems, the HLDB and the magnocellular preoptic nucleus provide cholinergic centrifugal innervation to the OB. The system also project broadly to other brain regions such as cortex and hippocampus. (El-Etri et al., 1999; Zaborszky et al., 1999; Matsutani and Yamamoto, 2008; Ma and Luo, 2012). It is currently unknown whether this system would provide specific regulation to the AOB. In the OB, cholinergic modulation sets the physiological state of attention and enhances odor discrimination (Smith and Araneda, 2010; Nunez-Parra et al., 2013; Devore et al., 2014; D’Souza and Vijayaraghavan, 2014; Rothermel et al., 2014; Smith et al., 2015; Bendahmane et al., 2016). Also, manipulation of the centrifugal cholinergic system results in altered olfactory-guided social and sexual related behavior (Smith et al., 2015). Because of the lack of detailed information about local cholinergic networks, cholinergic modulation in the AOB is generally considered to be mediated solely by the centrifugal innervation (Smith et al., 2015). However, it is unknown whether the activity of local cholinergic interneurons is influenced by the centrifugal projection. Furthermore, the main olfactory system also detects semiochemicals and plays an important role in regulating social and sexual behavior either directly or indirectly by guiding animals to stimulus sources (Keverne, 2004; Lin et al., 2004, 2007; Restrepo et al., 2004, 2006; Mandiyan et al., 2005; Spehr et al., 2006a,b; Fraser and Shah, 2014; López et al., 2014; Beny and Kimchi, 2016). This directional guidance by the main olfactory system is needed to bring animals in proximity to the stimuli to facilitate vomeronasal sensing of semiochemicals, especially nonvolatile pheromones that require animals contact the stimuli physically with their nose (Luo et al., 2003; Keverne, 2004; Restrepo et al., 2004; Kang et al., 2009; Martel and Baum, 2009; Slotnick et al., 2010). Therefore, alterations in social and sexual behavior resulting from manipulation of the centrifugal cholinergic system may involve local cholinergic networks in both the MOB and AOB. It will be interesting to determine the relationship between centrifugal projection and the local cholinergic networks.

Based on the distribution of the local cholinergic interneurons we identified in this study, we expect that in the aAOB, local cholinergic interneurons may be primarily involved in modulation of initial information processing. This is because most of the putative cholinergic cells are juxtaglomerular cells found in the aGL. In our previous study of cholinergic interneurons in the MOB, we found that a subpopulation of these interneurons expresses CB (Krosnowski et al., 2012). As expected, the GFP+ and CB+ cells are partially overlapped. Unlike in the GL of MOB, there are significantly more GFP+ cells than CB+ cells in the GL of the AOB. Most striking observation in the GL of the AOB is the difference in the GFP+ density between the aGL and pGL. Thus, cholinergic modulation by local juxtaglomerular cells may occur at a higher rate in the aGL than in the pGL. This distribution pattern is also distinct from that in the MOB, where the distribution of the cholinergic juxtaglomerular interneurons is relatively even throughout the GL (Krosnowski et al., 2012).

In our study, we also identify a large number of putative cholinergic neurons in the MCL with outstanding features. First, their distribution pattern is clearly dichotomous with a majority of them densely residing in the pMCL. Second, within the pMCL, the majority of these cell bodies are located in the outer region. From the immunolabeling and morphological examination, we conclude that most of these putative cholinergic neurons are non-glutamatergic neurons because of the negative immunoreactivity for the anti-GluR2/3 antibody and relatively smaller cell bodies compared to the surrounding mitral/tufted cells. In our published study of cholinergic interneurons in the MOB, we reported a set of cholinergic neurons in the external plexiform layer (EPL) with very long processes often running in parallel to the MCL for a long distance. The proximal regions of these long processes often do not branch (Krosnowski et al., 2012). The putative cholinergic cells we identified in the MCL of the AOB share some similar features. Unlike those in the EPL of the MOB, a small subset of the cholinergic cells in the pMCL shows GluR2/3-ir, similar to mitral/tufted cells. The GFP+ neurons in the MCL we observed also share some anatomical and morphological features with the “round projecting cells” identified in rats (Larriva-Sahd, 2008). Based on the Nissl-staining in rat AOB sections, Larriva-Sahd (2008) reported that a subset of neurons possesses long processes that bear varicosities and few spines in the region that is equivalent to the MCL of the AOB in mice. These cells are found more in the pAOB than the aAOB. Some of them have noticeable axons projecting to the LOT. Neurochemical property of these round cells in rats has not been reported. Because of their anatomical and morphological similarities, we believe the putative cholinergic neurons in the MCL of the mouse AOB identified in this study and the round projecting cells belong to the same cell type. Therefore, the presence of these neurons in the MCL of the AOB is conserved between mice and rats.

Under current classification, the mouse MCL of the AOB, which includes the EPL, houses multiple cell types. Recent reports have shown distinctive physiological features among a subset of MCL neurons (Gorin et al., 2016; Vargas-Barroso et al., 2016). In rats, Larriva-Sahd (2008) suggested that round cells are projecting neurons because their thin axonal processes extend to the LOT. Whether the putative pMCL cholinergic neurons in mouse are local or projection neurons remain to be determined. Despite their unknown physiological functions, our current findings are significant because the data clearly indicate regionally-distributed novel populations of local neurons that are likely integral to the dichotomous information processing in the AOB.

Another striking anatomical feature described in this study is the invagination of the nerve plexuses between the aAOB and pAOB, which is conserved between rats and mice. We further show that these nerve invaginations contain dense nerve fibers from local putative cholinergic neurons, most of which are located in the pMCL. We also establish that these nerve plexuses are within a small subset of glomeruli that also receive extensive glutamatergic innervation from mitral/tufted cells because of their positive labeling with antibodies against both VgluT2 and GluR2/3. From the immunolabeling of Gαi2, it is also clear that these glomeruli do not belong to the aAOB. This is in agreement with the observation that most of the GFP+ nerve fibers are from the pMCL neurons. This unique anatomical arrangement is very consistent among individual mice, which further implies a potentially significant physiological role of these glomeruli in the AOB information processing.

Our characterization of local cholinergic neurons in the AOB is significant because local interneurons are known to play an essential role in sensory signal processing (Luo et al., 2003; Castro et al., 2007; Peretto and Paredes, 2014). Similar to the MOB, various interneurons that are either GABAergic, or dopaminergic, or glutamatergic are present in the AOB (Mugnaini et al., 1984; Takami et al., 1992; Hayashi et al., 1993; Yokosuka, 2012). However, characterization of cholinergic interneurons has been challenging and the resulted in controversial understanding of these neurons in the AOB (Carson and Burd, 1980; Ojima et al., 1988; Ichikawa et al., 1997; Crespo et al., 1999). Several factors have contributed to this controversy. First, ChAT and VAChT function at nerve terminals where ACh molecules are synthesized, stored and released. From our previous study, the expression of these proteins in the cell bodies of bulbar interneurons is relatively weak as evident from immunolabeling (Krosnowski et al., 2012). Second, the AOB also receives strong centrifugal cholinergic innervation similar to the MOB, which often masks the local cholinergic network unless an additional marker such as GFP is used to visualize the cell bodies. We were able to circumvent this problem using the ChAT^(BAC)^-eGFP transgenic mouse line that provides strong GFP expression in the cell bodies. In our previous studies, we carefully characterized the cholinergic status of GFP+ non-neuronal chemosensory cells or neurons in this mouse line by using antibodies against ChAT and VAChT and have found GFP+ cells express both ChAT and VAChT (Ogura et al., 2010, 2011; Krosnowski et al., 2012). In this study, we observed uneven intensity of VAChT and ChAT labeling which is strong in the nerve fibers and barely detectable in the cell bodies (Figure 2). This result is similar to those found in the MOB. However in comparison to the MOB staining, the VAChT-ir and ChAT-ir appeared relatively weaker and were not present in all the GFP+ neurons of the AOB. Several factors, including the weak expression of these proteins, insufficient sensitivity of the labeling method, and ectopic expression might contribute to this weaker VAChT-ir and ChAT-ir signal. Additionally, temporally regulated expression of cholinergic markers in these neurons might also result in the weak or absent VAChT-ir and ChAT-ir in some GFP+ neurons of the AOB. For these reasons we consider that the GFP+ neurons found in this study to be putatively cholinergic. Future physiological and pharmacological studies are needed to further confirm their cholinergic status.

In conclusion, we have identified a large population of putative cholinergic neurons in the AOB and provided some characterization of their anatomical and morphological features and cell marker expression. Our data uncover the striking difference in the distribution of these neurons between the aAOB and pAOB, which is indicative of a potentially important role in dichotomous information processing in the AOB.

## Author Contributions

SM and KK performed experiments and data analysis. TO did some data analysis and editing the manuscript. SM also drafted a part of the manuscript. WL conceived, supervised the project and drafted most of the manuscript. All authors read, edited and approved the manuscript.

## Funding

This work was supported by NIH/National Institute on Deafness and Other Communication Disorders (NIDCD) DC009269 and DC012831 to WL.

## Conflict of Interest Statement

The authors declare that the research was conducted in the absence of any commercial or financial relationships that could be construed as a potential conflict of interest.
